# The emerging role of SARS-CoV-2 nonstructural protein 1 (nsp1) in epigenetic regulation of host gene expression

**DOI:** 10.1093/femsre/fuae023

**Published:** 2024-09-04

**Authors:** Konstantin I Ivanov, Haibin Yang, Ruixue Sun, Chunmei Li, Deyin Guo

**Affiliations:** Guangzhou National Laboratory, Guangzhou, 510320, China; Department of Microbiology, University of Helsinki, Helsinki, 00014, Finland; MOE Key Laboratory of Tropical Disease Control, Center for Infection and Immunity Studies (CIIS), School of Medicine, Shenzhen Campus of Sun Yat-Sen University, Sun Yat-Sen University, Shenzhen, 518107, China; Guangzhou National Laboratory, Guangzhou, 510320, China; MOE Key Laboratory of Tropical Disease Control, Center for Infection and Immunity Studies (CIIS), School of Medicine, Shenzhen Campus of Sun Yat-Sen University, Sun Yat-Sen University, Shenzhen, 518107, China; Guangzhou National Laboratory, Guangzhou, 510320, China; MOE Key Laboratory of Tropical Disease Control, Center for Infection and Immunity Studies (CIIS), School of Medicine, Shenzhen Campus of Sun Yat-Sen University, Sun Yat-Sen University, Shenzhen, 518107, China; State Key Laboratory of Respiratory Diseases, National Clinical Research Center for Respiratory Diseases, Guangzhou Institute of Respiratory Health, The First Affiliated Hospital of Guangzhou Medical University, Guangzhou, 510182, China

**Keywords:** coronavirus, nsp1, epigenetic silencing, heterochromatin, H3K9me2, DNA polymerase alpha

## Abstract

Infection with the severe acute respiratory syndrome coronavirus 2 (SARS-CoV-2) causes widespread changes in epigenetic modifications and chromatin architecture in the host cell. Recent evidence suggests that SARS-CoV-2 nonstructural protein 1 (nsp1) plays an important role in driving these changes. Previously thought to be primarily involved in host translation shutoff and cellular mRNA degradation, nsp1 has now been shown to be a truly multifunctional protein that affects host gene expression at multiple levels. The functions of nsp1 are surprisingly diverse and include not only the downregulation of cellular mRNA translation and stability, but also the inhibition of mRNA export from the nucleus, the suppression of host immune signaling, and, most recently, the epigenetic regulation of host gene expression. In this review, we first summarize the current knowledge on SARS-CoV-2-induced changes in epigenetic modifications and chromatin structure. We then focus on the role of nsp1 in epigenetic reprogramming, with a particular emphasis on the silencing of immune-related genes. Finally, we discuss potential molecular mechanisms underlying the epigenetic functions of nsp1 based on evidence from SARS-CoV-2 interactome studies.

## Teaser

This review examines the role of SARS-CoV-2 nonstructural protein 1 (nsp1) in epigenetic silencing of innate antiviral immune response genes and discusses potential molecular mechanisms underlying the nsp1-mediated epigenetic reprogramming.

## Introduction

The coronavirus disease of 2019 (COVID-19) is widely regarded as one of the deadliest viral diseases in human history, with more than 7 million deaths worldwide (according to WHO epidemiological data from April 2024). The underlying cause of COVID-19 is the infection with severe acute respiratory syndrome coronavirus 2 (SARS-CoV-2), a member of the genus *Betacoronavirus* of the *Coronaviridae* family of enveloped RNA viruses (Hu et al. 2021). The SARS-CoV-2 genome is a linear, nonsegmented, positive-sense ssRNA with a 5′-cap, a 5′-UTR, two large, partially overlapping open reading frames (ORF1a and ORF1b) encoding nonstructural proteins, several nested ORFs encoding structural and accessory proteins, a 3′-UTR, and a poly-A tail (V’Kovski et al. 2021, Malone et al. 2022). The nonstructural protein nsp1 is produced early in infection by cotranslational cleavage of the N-terminus of the ORF1a polyprotein (Snijder et al. 2003). The nsp1 amino acid sequence is well conserved between the two highly pathogenic human coronaviruses, SARS-CoV and SARS-CoV-2 (Yoshimoto 2020). The primary function of nsp1 is to shut off host gene expression, allowing the virus to overcome antiviral defense mechanisms that depend on *de novo* protein synthesis. The strategies by which nsp1 inhibits host gene expression are multipronged, targeting different critical cellular pathways and functions.

The first strategy is to inhibit host translation machinery and induce endonucleolytic cleavage and subsequent degradation of cellular mRNAs (Kamitani et al. 2006, 2009, Narayanan et al. 2008, Huang et al. 2011, Lokugamage et al. 2012, Tanaka et al. 2012, Finkel et al. 2021, Lapointe et al. 2021, Abaeva et al. 2023). A combination of cryo-EM and biochemical studies showed that nsp1 interacts with the ribosomal 40S subunit, blocking the mRNA entry channel (Schubert et al. 2020, Thoms et al. 2020, Yuan et al. 2020). The replacement of two positively charged amino acids, lysine 164 and histidine 165, in the C-terminal region of nsp1 with alanines resulted in the loss of binding to the 40S subunit and completely abolished the nsp1-mediated host gene shutoff (Narayanan et al. 2008, 2015, Tanaka et al. 2012). These residues are conserved in nsp1 proteins from SARS-CoV and SARS-CoV-2 (Simeoni et al. 2021), underscoring their functional importance. To induce cellular mRNA cleavage and subsequent degradation, nsp1 acts as a manganese- and calcium-dependent endonuclease even in the absence of any auxiliary factors (Salgueiro et al. 2024). However, binding of nsp1 to the ribosome stimulates endonuclease activity (Tardivat et al. 2023). Interestingly, a conserved SL1 hairpin found at the 5′ end of viral genomic and subgenomic RNAs (Miao et al. 2021, Bujanic et al. 2022, Sosnowski et al. 2022) protects them from nsp1-mediated cleavage and degradation (Finkel et al. 2021, Tardivat et al. 2023), suggesting a mechanism by which viral genes escape global translational shutoff.

The second strategy used by nsp1 to suppress host gene expression is to block the nuclear export of cellular mRNAs. This is achieved independently of nsp1 binding to the ribosome (Fisher et al. 2022) through a direct interaction between nsp1 and the mRNA nuclear export receptor NXF1 (Zhang et al. 2021a). Furthermore, nsp1 binding to nucleoporins (Gomez et al. 2019, Zhang et al. 2021a), which are the building blocks of the nuclear pore complex (NPC) (Kim et al. 2018, Yang et al. 2023), may alter the NPC structure and interfere with NXF1–mRNA docking at the NPC. The resulting nsp1-mediated inhibition of mRNA nuclear export reduces antiviral protein synthesis while increasing the availability of the host cell translation machinery to viral RNAs.

The third strategy by which nsp1 affects host gene expression is by antagonizing type I interferon (IFN-I) signaling, which promotes the activation of antiviral interferon-stimulated genes (ISGs). The expression of these genes is controlled by the JAK/STAT signaling pathway, which involves the phosphorylation of STAT1/STAT2 transcription factors to facilitate their nuclear translocation in a complex with a third transcription factor, IRF9 (Platanias 2005, Au-Yeung et al. 2013). Therefore, interfering with STAT phosphorylation provides a possible means for the virus to suppress ISG expression in infected cells. Indeed, nsp1 has been shown to inhibit the phosphorylation of STAT1 in cells stimulated with IFN-α (Wathelet et al. 2007, Xia et al. 2020). Thus, nsp1 has the ability to specifically block ISG expression by inhibiting the JAK/STAT signaling pathway at the level of STAT1 phosphorylation. Furthermore, nsp1 inhibits the upstream retinoic acid-inducible gene I (RIG-I) pathway, which connects the RIG-I-like receptor, a key sensor of viral infection, to type I interferon transcription (Xia et al. 2020). Taken together, the above findings indicate that nsp1 is a surprisingly multifunctional protein that helps the virus evade host defenses by interfering with various cellular processes and pathways. However, recent evidence suggests that, in addition to these functions, nsp1 is also directly involved in epigenetic regulation.

This review does not attempt to cover all aspects of how nsp1 affects host mRNA translation, stability, nuclear export, and innate immune responses, as these have already been thoroughly reviewed elsewhere (Yuan et al. 2021, Karousis 2024). Instead, we will focus on the emerging role of nsp1 in epigenetic regulation. We will first briefly outline how SARS-CoV-2 infection alters histone post-translational modifications (PTMs) and chromatin architecture in the host cell. We will then summarize the key findings supporting the central role of nsp1 in epigenetic reprogramming during SARS-CoV-2 infection. Finally, we will discuss potential molecular mechanisms underlying nsp1-mediated epigenetic silencing of antiviral immune-related genes.

### SARS-CoV-2 infection interferes with DNA and histone epigenetic modifications

Epigenetic modifications of nucleic acids (Chen et al. 2017a) and histones (Zhang et al. 2021b) allow the cell to quickly respond to various environmental and pathogenic stimuli by reprogramming gene transcription (Chen et al. 2017b, Zhang et al. 2020b, Fritz et al. 2022). While the cellular mechanisms underlying such epigenetic modifications play an important role in antiviral defense, viruses can also hijack them for their own benefit (Tsai and Cullen 2020). A growing body of evidence suggests that SARS-CoV-2 infection affects both DNA methylation and histone PTMs, which are the two fundamental epigenetic mechanisms that regulate gene expression.

Several studies have used circulating blood cells from COVID-19 patients to investigate how SARS-CoV-2 infection affects DNA methylation, an epigenetic modification associated with gene silencing (Newell-Price et al. 2000). Although blood cells are not the primary target of SARS-CoV-2, the virus has been shown to infect monocytes (Codo et al. 2020, Pontelli et al. 2022), monocyte-derived macrophages, dendritic cells (Zheng et al. 2021), T lymphocytes (Pontelli et al. 2022, Shen et al. 2022), and B lymphocytes (Pontelli et al. 2022). Depending on the blood cell type, the infection can be either productive or abortive, angiotensin-converting enzyme 2 (ACE2)-dependent or independent. Corley et al. (2021) identified multiple differentially methylated loci in the peripheral blood mononuclear cells (PBMCs) of severe COVID-19 patients. They showed that the DNA methylation signature associated with severe COVID-19 is characterized by hypermethylation of IFN-related genes and hypomethylation of inflammatory genes, supporting the hypothesis that SARS-CoV-2 hijacks the host epigenome to suppress antiviral immune responses and promote uncontrolled inflammation. In addition, the researchers discovered that DNA methylation aging clocks were accelerated in severe COVID-19 patients. Zhou et al. (2021) investigated genome-wide DNA methylation profiles in the whole blood of healthy subjects and COVID-19 patients with different levels of disease severity. They found that the SARS-CoV-2 infection caused a global reprogramming of DNA methylation in blood cells of infected patients. The authors linked the changes in DNA methylation to the regulation of inflammatory and immune-related genes, further supporting a clinically relevant role for this epigenetic mechanism. Other studies have also linked the differentially methylated DNA regions in peripheral blood cells of COVID-19 patients to genes involved in antiviral immune responses, leukocyte activity, and autoimmune diseases (Balnis et al. 2021, Castro de Moura et al. 2021, Konigsberg et al. 2021, Barturen et al. 2022). Using a mouse model of SARS-CoV-2 infection, Li et al. (2021a) identified multiple differentially methylated sites at gene promoters in the heart and kidney of infected animals. Notably, both organs exhibit SARS-CoV-2 tropism in humans (Liu et al. 2021). The study by Noguera-Castells et al. (2024) stands out for its focus on altered DNA methylation in the lung, which is the primary target of SARS-CoV-2 infection. The authors analysed lung autopsy samples from patients who died of COVID-19-related pneumonia to identify the DNA methylation signature associated with respiratory failure. The identified signature was enriched in genes related to inflammation, cell adhesion, and immune response, suggesting the clinical significance of epigenetic changes in DNA methylation during native SARS-CoV-2 lung infection. These results are particularly important because they show that SARS-CoV-2 has a universal effect on DNA methylation signatures not only in peripheral blood cells but also in lung epithelial cells, the primary target of infection. Finally, SARS-CoV-2 infection leaves long-term epigenetic traces in PBMCs or total leukocytes of recovered patients in the form of altered DNA methylation patterns (Balnis et al. 2022, Huoman et al. 2022, Nikesjo et al. 2022), suggesting a role for this modification in the persistence of postacute sequelae of COVID-19, also known as “long COVID.”

PTM of histones is another key epigenetic mechanism that regulates gene expression. There is growing evidence that SARS-CoV-2 infection has a profound effect on histone PTMs. Histone H3 lysine 27 acetylation (H3K27ac), a PTM associated with active enhancers, is one notable example. Wang et al. (2023) showed that infection of cultured ACE2-overexpressing A549 lung cells (A549-ACE2) with SARS-CoV-2 caused a global decrease in this active chromatin mark, which was associated with transcriptional repression of host genes involved in the antiviral immune response. At the same time, the authors found a specific increase in the active promoter mark histone 3 lysine 4 trimethylation (H3K4me3) at proinflammatory gene promoters, suggesting an epigenetic mechanism for the activation of proinflammatory cytokines observed in COVID-19 patients. Yang et al. (2022) analysed the profiles of H3K4me3 and the repressive mark histone 3 lysine 27 trimethylation (H3K27me3) in PBMCs from hospitalized COVID-19 patients. They found selective changes in H3K4me3 or H3K27me3 near the transcription start sites of several microRNAs that regulate immune and inflammatory responses. Leppkes et al. (2020) showed that blood serum of COVID-19 patients had increased levels of another epigenetic modification, citrullination of histone 3 (Cit-H3). They found that the increase in Cit-H3, an epigenetic mark of decondensed and transcriptionally active chromatin (Christophorou et al. 2014), was associated with increased proinflammatory cytokine expression and release of extracellular chromatin traps (NETs) by neutrophils during severe COVID-19. Yet another epigenetic PTM affected by SARS-CoV-2 infection is histone proteolytic cleavage. Histone cleavage is an emerging epigenetic mechanism whose role in regulating chromatin structure and gene expression is only beginning to be understood (Yi and Kim 2018). Huckriede et al. (2021) detected extracellular histone H3 in the plasma of hospitalized severe COVID-19 patients and showed that the H3 was often proteolytically cleaved. The authors hypothesized that the cleavage, most likely performed by neutrophil elastase at the N-terminal tail of H3, may be part of a yet-to-be-described NETosis-related regulatory mechanism. Although many of the above studies are limited by the use of *in vitro*-cultured lung cells or peripheral blood cells, which are not the primary target of SARS-CoV-2, there is a growing consensus that epigenetic modifications of DNA and histones play an important and clinically relevant role during SARS-CoV-2 infection. Nonetheless, research on coronavirus-induced epigenetic modifications is still in its early stages, leaving many questions unanswered. For example, the molecular mechanism(s) underlying the locus-specificity of virus-induced epigenetic modifications remain largely unknown. Without this knowledge, it would be difficult to understand how SARS-CoV-2 epigenetically silences antiviral immune response genes while activating others, such as those involved in inflammation. For information on enzymes, which may be involved in epigenetic modifications in the context of SARS-CoV-2 infection, we refer the reader to comprehensive reviews available elsewhere (Kgatle et al. 2021, Bhat et al. 2022, Foolchand et al. 2022, Dey et al. 2023).

### SARS-CoV-2 infection rearranges host chromatin architecture

Dynamic changes in chromatin architecture play an important role in gene regulation during development, normal physiology, and disease (Pombo and Dillon 2015). Several viruses are known to interfere with epigenetic mechanisms that regulate host chromatin architecture to establish persistent infection and evade host defenses (Tsai and Cullen 2020, Friedman et al. 2022). One such virus is SARS-CoV-2. Ho et al. (2021) used the Hi-C chromosome conformation capture technique to demonstrate that SARS-CoV-2 infection causes extensive rearrangement of chromatin topology in cultured A549-ACE2 cells. They showed that the infection caused a significant redistribution of active (A) and inactive (B) chromatin compartments in infected cells. This redistribution was characterized by the shortening of long A and B chromatin domains and their conversion into mixed A–B subdomains. In addition, the authors performed chromatin immunoprecipitation and sequencing (ChIP-seq) analysis for H3K27ac, an epigenetic mark of active enhancers, to show the association of SARS-CoV-2-induced chromatin restructuring with changes in transcriptional activity. These changes affected genes involved in proinflammatory and antiviral immune responses, suggesting that they are the targets of virus-induced epigenetic reprogramming. A more recent study by Wang et al. (2023) found that SARS-CoV-2 infection globally restructures host chromatin in A549-ACE2 cells by weakening compartment A, A–B mixing, and reducing contacts within self-interacting topologically associating domains (TADs). Importantly, genomic loci containing ISGs had fewer intra-TAD promoter–enhancer contacts and less H3K27ac deposition in SARS-CoV-2-infected cells compared to mock-infected controls. The findings of Wang et al. (2023) were recently confirmed in a study employing polymer physics-based molecular modeling. The study showed that SARS-CoV-2 infection caused TAD rearrangements at ISG genomic loci by reducing the specificity and structural stability of regulatory contacts (Chiariello et al. 2023). In addition, several independent studies have found that SARS-CoV-2 induces large-scale chromatin remodeling in COVID-19 patients’ PBMCs or sorted CD14^+^ monocytes, affecting the expression of immune pathway-related genes. Such chromatin remodeling was influenced by the stage and severity of the disease, as well as the age of the patient (Zheng et al. 2020, Li et al. 2021b, Brauns et al. 2022, Giroux et al. 2022). Furthermore, SARS-CoV-2 infection induced extensive and long-lasting changes in chromatin accessibility and transcriptional profiles of convalescent COVID-19 patients (You et al. 2021, Cheong et al. 2023). These changes may represent an epigenetic memory of a previous immune challenge by SARS-CoV-2 that progeny monocytes inherit from their progenitors (Cheong et al. 2023). However, this epigenetic immune memory, also known as trained immunity, may not only protect against reinfection but also contribute to the long-term clinical sequelae of COVID-19. Taken together, these findings suggest that epigenetic mechanisms involving genome-wide chromatin remodeling contribute to various immune and inflammatory phenomena observed in COVID-19 patients. We are making gradual progress in understanding these mechanisms, but there are still many unanswered questions. Among these questions, one is particularly important: which viral factors are involved in these epigenetic mechanisms?

### Unveiling the role of nsp1 as an epigenetic regulator

Lee et al. (2023) employed Assay for Transposase-Accessible Chromatin using sequencing (ATAC-seq) to assess genome-wide changes in chromatin accessibility in cultured VeroE6 cells transiently transfected with plasmids encoding all SARS-CoV-2 proteins (Lee et al. 2023). Of the 29 proteins tested, only two, nsp1 and, somewhat unexpectedly, spike, induced widespread changes in chromatin accessibility. The fact that nsp1 and spike, even when expressed alone, were able to globally modify the chromatin accessibility landscape suggested an active role for these proteins in genome-wide chromatin restructuring. However, the authors found significant differences between the chromatin accessibility profiles of nsp1- and spike-expressing cells. First, nsp1 had a more global effect on chromatin accessibility, with at least twice as many ATAC-seq peaks induced by nsp1 compared to those induced by spike. The number of both positive and negative ATAC-seq peaks was higher in nsp1-expressing cells, indicating that nsp1 generated more regions with increased and decreased chromatin accessibility than spike. Second, and most importantly, the chromatin accessibility signatures of nsp1 and spike were significantly different, with only a minor overlap. One explanation for this is that nsp1 promotes a different transcriptional program than spike, possibly through a different mechanism. Furthermore, the authors showed a significant and positive correlation between changes in chromatin accessibility induced by nsp1 and those observed during SARS-CoV-2 infection, confirming that ATAC-seq data obtained with individually expressed nsp1 is physiologically relevant.

The simplest and most straightforward explanation for the widespread nsp1-induced changes in chromatin accessibility is that nsp1, a known translational repressor, inhibits the translation of mRNAs encoding master transcriptional and epigenetic regulators. However, a recent study by Anastasakis et al. (2024) provides another explanation: the existence of an nsp1-mediated, translation-independent epigenetic mechanism. The study combined several high-throughput approaches to investigate how nsp1 affects the expression of host immune-related genes. First, the authors used RNA-seq in transiently transfected A549 cells to show that nsp1 expression significantly reduced the steady-state mRNA levels of host genes involved in innate immunity and antiviral defense. The analysis was performed within a narrow time window of 16–24 hours post-transfection when nsp1 expression levels were still low, mimicking the situation early in SARS-CoV-2 infection. Within this time window, nsp1 expression levels are still insufficient to suppress host cell mRNA translation through stoichiometric binding to the host translation machinery. To test this experimentally, Anastasakis et al. (2024) used Ribo-seq, also known as ribosome profiling, to determine how nsp1 expression affects the efficiency of host gene translation. They found that early after transfection, nsp1 had no effect on the translation efficiency of the downregulated genes identified by RNA-seq, implying that inhibition of translation could not explain the reduced expression of antiviral immune-related genes. This prompted the authors to investigate whether nsp1 regulates the process of host gene transcription itself, rather than translation or stability of the resulting transcripts. They performed RNA polymerase II (Pol II) ChIP-seq and found that nsp1-induced changes in Pol II occupancy correlated well with changes in mRNA levels from RNA-seq data. Furthermore, they showed that nsp1 increased the deposition of the repressive histone H3 lysine 9 dimethylation mark (H3K9me2) at the silenced immune-related genomic loci. Finally, they demonstrated that specific pharmacological inhibition of the H3K9me2 methyltransferase G9a, alternatively known as euchromatic histone lysine *N*-methyltransferase 2, restored the expression of antiviral immune-related genes suppressed by nsp1 and significantly inhibited SARS-CoV-2 infection, resulting in an ~10-fold reduction in viral load. Thus, the results of Anastasakis et al. (2024) suggest that nsp1 inhibits host antiviral defenses not only at the cellular mRNA level, but also at the epigenetic level. Specifically, nsp1 induces epigenetic silencing of immune-related genes through the G9a-mediated deposition of the signature heterochromatin mark H3K9me2 at the corresponding genomic loci.

### Putative mechanisms of nsp1-mediated epigenetic regulation

The molecular mechanism by which nsp1 promotes repressive histone methylation to silence immune-related genes is currently unknown. However, the identification of host factors interacting with nsp1 suggests several possibilities, which are discussed below.

#### Direct nsp1 association with chromatin

One possible mechanism by which nsp1 may induce epigenetic reprogramming is by entering the nucleus and interacting directly with the chromatin remodeling machinery. Indeed, several host proteins identified as nsp1 interactors in high-throughput studies are known to be associated with chromatin (Li et al. 2023). Although SARS-CoV-2 nsp1 is a predominantly cytoplasmic protein, several independent studies have shown that it also localizes to the nucleus in plasmid-transfected cells (Gordon et al. 2020a, Zhang et al. 2020a, 2021a, Lee et al. 2021, Shemesh et al. 2021). According to our unpublished observations, the nuclear-enriched fraction of 293T cells contains up to 10% of total cellular SARS-CoV-2 nsp1 at later time points after transfection. In SARS-CoV-infected cells, a small but distinct proportion of nsp1 is detected in the nucleus from 9 hours postinfection (Prentice et al. 2004). Since SARS-CoV nsp1 has more than 90% sequence similarity with its SARS-CoV-2 ortholog (Yoshimoto 2020), these two proteins may share a common subcellular localization. However, other studies have found that SARS-CoV-2 nsp1 is almost exclusively located in the cytoplasm of infected cells (Gordon et al. 2020a, Shi et al. 2022). In any case, the functional significance of nsp1nuclear localization remains unknown. Although this seems to be an attractive possibility, there are no published observations to support the hypothesis that nsp1 is directly bound to nucleosomes. Thus, whether nsp1 exerts its epigenetic function through the direct interaction with chromatin-associated machinery remains, so far, uncertain.

#### Nsp1 interaction with PRRC2B

In an alternative mechanism to association with chromatin, nsp1 may act indirectly to induce epigenetic silencing of immune-related genes by interacting with specific regulatory proteins. Nsp1 binding to such regulatory proteins may take place in the cytoplasm, thereby obviating the need for nsp1 to enter the nucleus. Anastasakis et al. (2024) focused on one such protein, PRRC2B, known to localize to both nucleus and cytoplasm (Thul et al. 2017, Jiang et al. 2023) and to interact with nsp1 (Samavarchi-Tehrani et al. 2020). Importantly, the nsp1-inactivating double mutation K164A/H165A reduced its affinity to PRRC2B (Samavarchi-Tehrani et al. 2020), suggesting a functional role for this protein–protein interaction. What makes PRRC2B special among other nsp1 interactors is that it also interacts with the H3K9me2 writer G9a (Rual et al. 2005). Consistent with a potential role for PRRC2B in regulating repressive histone methylation through binding to G9a, Anastasakis et al. (2024) showed that siRNA-mediated knockdown of PRRC2B reversed the nsp1-induced silencing of immune-related genes. Taken together, these results suggest that the mechanism of nsp1-mediated epigenetic silencing of immune-related genes may involve PRRC2B. Further experiments are required to validate this proposed mechanism.

#### Nsp1 interaction with Pol α

Another candidate mechanism for the nsp1-mediated epigenetic gene silencing involves the interaction nsp1 with DNA polymerase alpha, also known as Pol α. This four-subunit enzyme is best known for synthesizing a short RNA primer and extending it by ~20 deoxyribonucleotides during DNA replication (Pellegrini 2012). However, Pol α also performs other functions, as discussed below. The four subunits of Pol α (POLA1, POLA2, PRIM1, and PRIM2), collectively referred to as the primosome, were originally identified as nsp1 binding partners in a high-throughput proteomic study by Gordon et al. (2020b). The association of nsp1 with Pol α subunits has since been confirmed in many other studies of the SARS-CoV-2–host interactome (Li et al. 2023). The consistent identification of Pol α as a binding partner of nsp1 prompted Kilkenny et al. (2022) to solve the cryo-EM structure of this multiprotein complex. The structure revealed that nsp1 interacts with Pol α through the inactive exonuclease domain of the POLA1 catalytic subunit. Consistent with this observation, the authors showed that binding to nsp1 does not affect the ability of Pol α to synthesize primers for DNA replication. Furthermore, the interaction between nsp1 and Pol α involves the conserved middle domain of nsp1, but not the C-terminal region responsible for ribosome targeting and inhibition of mRNA translation (Schubert et al. 2020, Thoms et al. 2020, Lapointe et al. 2021). Thus, the results of Kilkenny et al. (2022) suggest that the interaction between nsp1 and Pol α is unlikely to affect either host mRNA translation or DNA replication.

In addition to its role in DNA replication, Pol α has been implicated in heterochromatin maintenance (Li and Zhang 2012) and modulation of the interferon response (Starokadomskyy et al. 2021). Before discussing these functions in more detail, it is important to note that all Pol α subunits are conserved across eukaryotes (Shultz et al. 2007), suggesting a shared function in diverse organisms. Several lines of evidence indicate that Pol α is involved in epigenetic inheritance of heterochromatin during DNA replication-coupled nucleosome reassembly. Studies in fission yeast found that mutations in the catalytic subunit of Pol α reduced the recruitment of Swi6, a yeast ortholog of the animal heterochromatin protein 1 (HP1), to heterochromatic loci, resulting in their activation (Ahmed et al. 2001, Nakayama et al. 2001). In addition, these studies showed that Swi6 directly interacts with the C-terminal part of the catalytic subunit of Pol α. Similar results were obtained in plants, where the catalytic subunit of Pol α was found to physically and genetically interact with the Swi6 plant ortholog LHP1 (like HP1) (Barrero et al. 2007). The primary function of HP1 and its orthologs is to specifically recognize the repressive H3K9me2/3 marks and promote epigenetic gene silencing through heterochromatin formation (Bannister et al. 2001, Lachner et al. 2001). However, in addition to being an H3K9me2/3 reader, HP1 maintains the protein stability of H3K9 methyltransferases, including G9a, by tethering them to chromatin (Maeda and Tachibana 2022). Consequently, the recruitment of HP1 by Pol α during DNA replication may promote the deposition of repressive H3K9 methylation marks on newly assembled nucleosomes. Consistent with this possibility, a mutation in the catalytic subunit of Pol α reduced promoter-associated H3K9me2 levels in *Arabidopsis* (Liu et al. 2010). Thus, it can be hypothesized that nsp1 represses the transcription of host immune-related genes, at least in part, by exploiting the physiological mechanism that propagates the silenced chromatin state during host cell division. This mechanism may involve the interaction of nsp1 with Pol α and operate through the Pol α-HP1-G9a-H3K9me2 axis. The presence of Pol α in both the nucleus and cytoplasm (Brown et al. 1981, Thul et al. 2017) suggests that the interaction of nsp1 with Pol α may occur not only in the nuclear compartment but also in the cytoplasm, eliminating the strict requirement for nsp1 to enter the nucleus.

**Figure 1.**
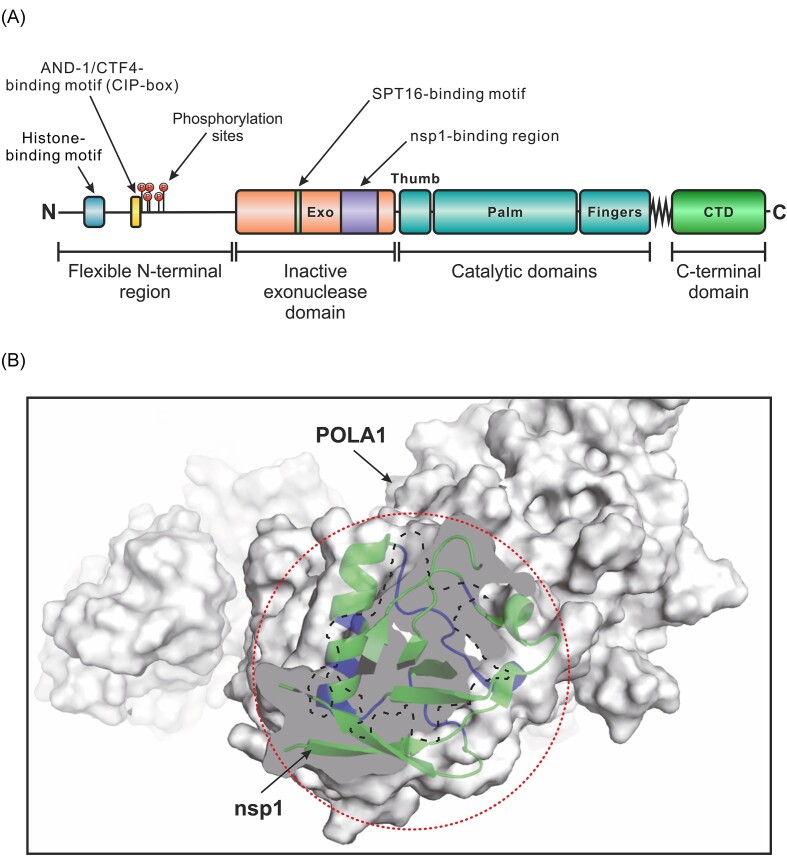
Specific interaction between nsp1 and the catalytic subunit of DNA polymerase alpha (POLA1) may affect its epigenetic functions. (A) Domain map of POLA1 showing the nsp1-binding region and sequence motifs involved in epigenetic inheritance. Based on the diagram by Nasheuer and Onwubiko (2023). (B) The interaction between nsp1 and POLA1 may prevent regulatory proteins from accessing the flexible N-terminal region of POLA1. POLA1 is shown as a gray space-filling model. A cross-section of the catalytically inactive POLA1 exonuclease domain is shown in solid gray. The contact area between POLA1 and nsp1 is highlighted with the dashed line. Nsp1 is drawn as ribbons, with POLA1-interacting structural elements in purple and the rest in green. The dotted circle indicates a potential steric clash between nsp1 and binding partners of the flexible N-terminal region of POLA1. The schematic rendering of the nsp1-POLA1 complex is based on the published cryo-EM structure by Kilkenny et al. (2022).

Given the role of Pol α in heterochromatin inheritance, it is perhaps not surprising that the N-terminal region of POLA1 contains a conserved histone-binding motif. This motif interacts with histones H2A–H2B (Evrin et al. 2018) and H3–H4 (Li et al. 2020) to maintain transcriptional silencing at heterochromatic regions and facilitate the transfer of parental histones to lagging DNA strands. In addition, the nearby CIP-box sequence interacts with the trimeric scaffold protein AND-1/CTF4 (Simon et al. 2014), which is dispensable for DNA replication but is also involved in parental histone transfer (Evrin et al. 2018, Gan et al. 2018). At least five phosphorylation sites, four of which are proline-directed, were identified immediately downstream of the CIP-box sequence (Hornbeck et al. 2015), suggesting that phosphorylation-induced conformational changes (Wulf et al. 2005, Gurung et al. 2023) could regulate POLA1-mediated epigenetic mechanisms. Another regulatory hotspot in POLA1 is the first glycine residue in the conserved GPCWL motif, which is critical for binding to the SPT16 subunit of the histone chaperone FACT (Zhou and Wang 2004). Notably, most of the above sequences are located in the N-terminal region of POLA1, which lacks a defined secondary structure. This flexible region sits immediately upstream of the catalytically inactive POLA1 nuclease domain, which contains the nsp1 binding site (Fig. 1A). Therefore, it can be hypothesized that nsp1 promotes POLA1-mediated epigenetic silencing by preventing regulatory proteins, including enzymes involved in PTMs, from accessing their binding sites in the flexible N-terminal region of POLA1. Consistent with this possibility, we found a possible steric clash between POLA1-bound nsp1 and the flexible N-terminus of POLA1 in the cryo-EM structure published by Kilkenny et al. (2022) (Fig. 1B).

Interestingly, SARS-CoV-2 infection upregulated all four subunits of Pol α at both the mRNA and protein levels (Puray-Chavez et al. 2021), suggesting the existence of a feedback mechanism that enhances the effects of Pol α during infection. Considering the role of POLA1 in epigenetic gene silencing through the interaction with HP1 and deposition of H3K9me2, it is tempting to speculate that SARS-CoV-2-induced overexpression of Pol α could promote epigenetic repression of antiviral immune-related genes in infected cells. Support for this possibility comes from research on X-linked reticulate pigmentary disorder (XLPDR), an orphan genetic disease caused by mis-splicing mutations in the *POLA1* gene resulting in partial POLA1 protein deficiency (Starokadomskyy et al. 2021). The reduced abundance of normally spliced *POLA1* transcripts causes a dramatic increase in the expression of ISGs (Starokadomskyy et al. 2019), leading to the development of an autoinflammatory disease in XLPDR patients. These findings suggest that sufficient availability of functional Pol α is essential for the tight transcriptional control of ISGs. Such transcriptional control can be brought about through an epigenetic mechanism involving repressive histone methylation and heterochromatin formation. An alternative, but not mutually exclusive, mechanism may involve dampening of antiviral nucleic acid sensors by Pol α-synthesized cytosolic RNA/DNA hybrids (Starokadomskyy et al. 2016). Thus, genetic evidence from human disease suggests that Pol α-mediated mechanism(s) control the expression of antiviral immune-related genes under normal physiological conditions. During SARS-CoV-2 infection, nsp1 may exploit these mechanisms to prevent immune-related genes from activating. One way how this can be achieved is through the direct interaction between nsp1 and Pol α, leading to the deposition of repressive H3K9me2 marks at the corresponding genomic loci and the formation of heterochromatin. Whether this speculative epigenetic mechanism actually exists, however, awaits further study. A complementary possibility is that nsp1 silences immune-related genes by interfering with the Pol α-mediated synthesis of immunogenic RNA/DNA hybrids in the cytoplasm. However, this scenario is less likely because the primer synthesis activity of Pol α is not affected by its interaction with nsp1 (Kilkenny et al. 2022).

### Synergism between nsp1-mediated inhibition of STAT phosphorylation and deposition of H3K9me2 on chromatin

**Figure 2.**
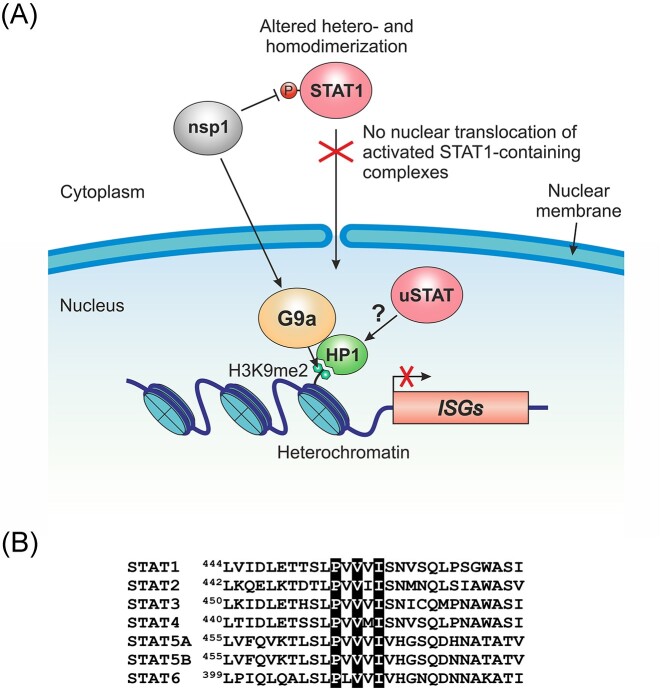
Nsp1 promotes transcriptional and epigenetic silencing of immune-related genes. (A) A model describing the role of nsp1 in ISG repression. Nsp1 antagonizes type I IFN signaling by inhibiting STAT1 phosphorylation in the cytoplasm. As a result, STAT1 is unable to form activated dimers with STAT2, preventing them from translocating into the nucleus and inducing ISG expression. In addition, nsp1 promotes G9a-mediated deposition of the repressive chromatin mark H3K9me2 at immune-related genomic loci through a yet unknown mechanism. The H3K9me2 mark is specifically recognized by HP1, promoting facultative heterochromatin formation. Nuclear unphosphorylated STATs (uSTATs), such as uSTAT3, may stabilize heterochromatin by specifically interacting with HP1. (B) All human STAT transcription factors contain the HP1-binding motif PxVxL/I (highlighted in black). Shown is an amino acid sequence alignment of the regions surrounding the HP1-binding motif in seven members of the human STAT protein family.

As mentioned above, nsp1 can repress ISG expression by inhibiting the phosphorylation of the transcription factor STAT1 in the cytoplasm. Reduced phosphorylation prevents STAT1 from entering the nucleus, recognizing specific enhancers, and activating ISG transcription. As we now know, the second way how nsp1 promotes ISG silencing is by adding the repressive mark H3K9me2 at the corresponding genomic loci (Fig. 2A). Since H3K9me2 prevents gene expression by reducing chromatin accessibility to transcription factors (Padeken et al. 2022), these two nsp1-mediated mechanisms are likely to be synergistic rather than mutually exclusive. Furthermore, these mechanisms may promote immune evasion in conjunction with other canonical nsp1-mediated mechanisms, such as translational repression, mRNA degradation, and inhibition of mRNA export from the nucleus.

Nuclear translocation of phosphorylated STAT (pSTAT) is an integral part of the canonical JAK/STAT pathway, which plays a central role in the IFN-mediated antiviral response. In the canonical pathway, unphosphorylated STAT transcription factors (uSTATs) are inactive in the cytoplasm and require phosphorylation for activation and nuclear translocation. However, a pool of uSTATs, including uSTAT1 (Meyer et al. 2002), is also present in the nucleus of unstimulated cells. These nuclear uSTAT proteins are involved in noncanonical STAT functions, which include basal transcriptional repression (Awasthi et al. 2021). All STAT transcription factors contain the conserved PxVxL/I motif in their DNA-binding domain (Fig. 2B). This motif is specifically recognized by the chromo shadow domain of the HP1 protein (Smothers and Henikoff 2000, Lechner et al. 2005, Brower-Toland et al. 2007, Xu et al. 2023). Since HP1 is the key structural component of heterochromatin and the reader of H3K9me2/3 (Canzio et al. 2014), its interaction with nuclear uSTATs promotes heterochromatin stability (Majoros et al. 2017). Although this phenomenon is best studied for uSTAT3 (Dutta et al. 2020), uSTAT5A (Hu et al. 2013), and *Drosophila* uSTAT92E (Shi et al. 2008), there is evidence that uSTAT1 also acts as a transcriptional repressor of specific genes in unstimulated cells (Zimmerman et al. 2012). Consistent with the role of nuclear uSTAT in maintaining heterochromatin stability, activation of the canonical JAK/STAT pathway shifts the balance toward pSTAT, causing heterochromatin disruption (Shi et al. 2006, Li 2008). Similarly, the ability of nsp1 to inhibit STAT1 phosphorylation and thus prevent the activation of the canonical JAK/STAT pathway may tip the balance in favor of heterochromatin stabilization (Fig. 2A). Such heterochromatin stabilization could be mediated, for example, by nuclear uSTAT3. Although STAT3 is activated by IFN-I stimulation in a manner similar to STAT1 and STAT2, it has the opposite (inhibitory) effect on the canonical IFN-I response by sequestering activated STAT1 into STAT1:STAT3 heterodimers (Ho and Ivashkiv 2006, Tsai et al. 2019). Consistent with the role of STAT3 as a suppressor of the IFN-I response, nuclear uSTAT3 promotes heterochromatin formation by binding to HP1 (Dutta et al. 2020). Nsp1 could stimulate this process by inhibiting STAT1 phosphorylation, effectively blocking the canonical JAK/STAT pathway, and thus preventing ISG transcriptional activation. Collectively, the above studies suggest that nsp1 employs several distinct but overlapping transcriptional and epigenetic strategies to effectively silence the expression of antiviral immune-related genes. These strategies are likely to be synergistic and involve viral interference with histone and transcription factor PTMs.

### Nsp1 sequence variation and epigenetic regulation

Although nsp1 is one of the least conserved nonstructural proteins among coronaviruses (Maurina et al. 2023), it is highly conserved among SARS-CoV-2 variants (Ghaleh et al. 2023, Kandwal and Fayne 2023). This suggests that the SARS-CoV-2 nsp1 sequence is well optimized, with a low tolerance for amino acid substitutions. On the other hand, the low sequence similarity of nsp1 among coronaviruses indicates that it is a hotspot of genetic variation, which allows the virus to adapt to different hosts, cell types, tissues, and other environmental factors. One possible strategy for the virus to achieve such adaptation is to selectively modify host gene expression via epigenetic reprogramming. Thus, it would be interesting to see whether the variation in nsp1 sequences across coronaviruses reflects the diversity of nsp1-mediated epigenetic mechanisms.

## Conclusions

Through millions of years of evolution, viruses and their hosts have been locked in an endless arms race, trying to stay one step ahead of each other. This arms race takes many forms and dimensions, with each side employing a variety of strategies to gain an advantage. However, viruses and their hosts differ fundamentally in one critical aspect of this arms race. Due to their compact genomes, viruses often lack the ability to encode dedicated proteins to perform specific functions, forcing them to rely on multifunctional proteins. The nsp1 protein of SARS-CoV-2 is an excellent example of how a single viral protein can perform a variety of functions to evade host immune defenses. To date, the most extensively studied function of nsp1 is to shut off cellular mRNA translation and promote its degradation. Furthermore, nsp1 blocks nuclear export of mRNA and inhibits the activation of IFN signaling. However, new evidence suggests that nsp1 is functionally even more complex than previously thought. We now know that nsp1 promotes epigenetic silencing of antiviral immune-related genes through the G9a-mediated deposition of the repressive mark H3K9me2 on the chromatin. This discovery not only adds another layer to our understanding of how nsp1 performs its multiple functions, but also raises new questions. A key open question is how exactly nsp1 induces H3K9me2-based heterochromatin formation. It is particularly important to identify the host factors involved and determine whether nsp1 performs its epigenetic function directly in the nucleus or indirectly through other proteins that shuttle between the cytoplasm and the nucleus. Another critical question is what determines the specificity of nsp1-mediated epigenetic silencing for immune-related gene loci. Despite intriguing hints from interactome studies, the above putative mechanisms underlying nsp1-mediated epigenetic silencing are, at best, speculative and require experimental validation. A better understanding of these mechanisms may lead to the development of novel nsp1-specific inhibitors, thus paving the way for new treatments for SARS-CoV-2 as well as other circulating and emerging coronavirus infections. As discussed above, virus-induced epigenetic reprogramming of host gene expression may contribute to the long-term effects of SARS-CoV-2 infection, also known as the “long COVID syndrome.” It would be interesting to know whether the nsp1-induced epigenetic changes play a role in the development of this heterogeneous syndrome with diverse clinical manifestations.

## References

[bib1] Abaeva IS, Arhab Y, Miscicka A et al. In vitro reconstitution of SARS-CoV-2 Nsp1-induced mRNA cleavage reveals the key roles of the N-terminal domain of Nsp1 and the RRM domain of eIF3g. Genes Dev. 2023;37:844–60. 37821106 10.1101/gad.350829.123 PMC10620056

[bib2] Ahmed S, Saini S, Arora S et al. Chromodomain protein Swi6-mediated role of DNA polymerase alpha in establishment of silencing in fission yeast. J Biol Chem. 2001;276:47814–21. 11581276 10.1074/jbc.M109186200

[bib3] Anastasakis DG, Benhalevy D, Cuburu N et al. Epigenetic repression of antiviral genes by SARS-CoV-2 NSP1. PLoS One. 2024;19:e0297262. 38277395 10.1371/journal.pone.0297262 PMC10817131

[bib4] Au-Yeung N, Mandhana R, Horvath CM. Transcriptional regulation by STAT1 and STAT2 in the interferon JAK-STAT pathway. JAKSTAT. 2013;2:e23931. 24069549 10.4161/jkst.23931 PMC3772101

[bib5] Awasthi N, Liongue C, Ward AC. STAT proteins: a kaleidoscope of canonical and non-canonical functions in immunity and cancer. J Hematol Oncol. 2021;14:198. 34809691 10.1186/s13045-021-01214-y PMC8607625

[bib6] Balnis J, Madrid A, Hogan KJ et al. Blood DNA methylation and COVID-19 outcomes. Clin Epigenet. 2021;13:118. 10.1186/s13148-021-01102-9 PMC8148415 34034806

[bib7] Balnis J, Madrid A, Hogan KJ et al. Persistent blood DNA methylation changes one year after SARS-CoV-2 infection. Clin Epigenet. 2022;14:94. 10.1186/s13148-022-01313-8 PMC9308917 35871090

[bib8] Bannister AJ, Zegerman P, Partridge JF et al. Selective recognition of methylated lysine 9 on histone H3 by the HP1 chromo domain. Nature. 2001;410:120–4. 11242054 10.1038/35065138

[bib9] Barrero JM, Gonzalez-Bayon R, del Pozo JC et al. INCURVATA2 encodes the catalytic subunit of DNA polymerase alpha and interacts with genes involved in chromatin-mediated cellular memory in *Arabidopsis thaliana*. Plant Cell. 2007;19:2822–38. 17873092 10.1105/tpc.107.054130 PMC2048701

[bib10] Barturen G, Carnero-Montoro E, Martinez-Bueno M et al. Whole blood DNA methylation analysis reveals respiratory environmental traits involved in COVID-19 severity following SARS-CoV-2 infection. Nat Commun. 2022;13:4597. 35933486 10.1038/s41467-022-32357-2 PMC9357033

[bib11] Bhat S, Rishi P, Chadha VD. Understanding the epigenetic mechanisms in SARS CoV-2 infection and potential therapeutic approaches. Virus Res. 2022;318:198853. 35777502 10.1016/j.virusres.2022.198853 PMC9236910

[bib12] Brauns E, Azouz A, Grimaldi D et al. Functional reprogramming of monocytes in patients with acute and convalescent severe COVID-19. JCI Insight. 2022;7. 10.1172/jci.insight.154183. PMC9090263 35380990

[bib13] Brower-Toland B, Findley SD, Jiang L et al. Drosophila PIWI associates with chromatin and interacts directly with HP1a. Genes Dev. 2007;21:2300–11. 17875665 10.1101/gad.1564307 PMC1973144

[bib14] Brown M, Bollum FJ, Chang LM. Intracellular localization of DNA polymerase alpha. Proc Natl Acad Sci USA. 1981;78:3049–52. 7019918 10.1073/pnas.78.5.3049 PMC319497

[bib15] Bujanic L, Shevchuk O, von Kugelgen N et al. The key features of SARS-CoV-2 leader and NSP1 required for viral escape of NSP1-mediated repression. RNA. 2022;28:766–79. 35232816 10.1261/rna.079086.121 PMC9014875

[bib16] Canzio D, Larson A, Narlikar GJ. Mechanisms of functional promiscuity by HP1 proteins. Trends Cell Biol. 2014;24:377–86. 24618358 10.1016/j.tcb.2014.01.002 PMC4077871

[bib17] Castro de Moura M, Davalos V, Planas-Serra L et al. Epigenome-wide association study of COVID-19 severity with respiratory failure. EBioMedicine. 2021;66:103339. 33867313 10.1016/j.ebiom.2021.103339 PMC8047083

[bib18] Chen Y, Hong T, Wang S et al. Epigenetic modification of nucleic acids: from basic studies to medical applications. Chem Soc Rev. 2017a;46:2844–72. 28352906 10.1039/c6cs00599c

[bib19] Chen Z, Li S, Subramaniam S et al. Epigenetic regulation: a new frontier for biomedical engineers. Annu Rev Biomed Eng. 2017b;19:195–219. 28301736 10.1146/annurev-bioeng-071516-044720

[bib20] Cheong JG, Ravishankar A, Sharma S et al. Epigenetic memory of coronavirus infection in innate immune cells and their progenitors. Cell. 2023;186:3882–902. 37597510 10.1016/j.cell.2023.07.019 PMC10638861

[bib21] Chiariello AM, Abraham A, Bianco S et al. Multiscale modelling of chromatin 4D organization in SARS-CoV-2 infected cells. Nat Commun. 2023;15:4014. 10.1038/s41467-024-48370-6 PMC11091192 38740770

[bib22] Christophorou MA, Castelo-Branco G, Halley-Stott RP et al. Citrullination regulates pluripotency and histone H1 binding to chromatin. Nature. 2014;507:104–8. 24463520 10.1038/nature12942 PMC4843970

[bib23] Codo AC, Davanzo GG, Monteiro LB et al. Elevated glucose levels favor SARS-CoV-2 infection and monocyte response through a HIF-1alpha/glycolysis-dependent axis. Cell Metab. 2020;32:498–9. 32877692 10.1016/j.cmet.2020.07.015 PMC7462530

[bib24] Corley MJ, Pang APS, Dody K et al. Genome-wide DNA methylation profiling of peripheral blood reveals an epigenetic signature associated with severe COVID-19. J Leukoc Biol. 2021;110:21–26. 33464637 10.1002/JLB.5HI0720-466R PMC8013321

[bib25] Dey A, Vaishak K, Deka D et al. Epigenetic perspectives associated with COVID-19 infection and related cytokine storm: an updated review. Infection. 2023;51:1603–18. 36906872 10.1007/s15010-023-02017-8 PMC10008189

[bib26] Dutta P, Zhang L, Zhang H et al. Unphosphorylated STAT3 in heterochromatin formation and tumor suppression in lung cancer. BMC Cancer. 2020;20:145. 32087696 10.1186/s12885-020-6649-2 PMC7036253

[bib27] Evrin C, Maman JD, Diamante A et al. Histone H2A-H2B binding by Pol alpha in the eukaryotic replisome contributes to the maintenance of repressive chromatin. EMBO J. 2018;37. 10.15252/embj.201899021. PMC6166128 30104407

[bib28] Finkel Y, Gluck A, Nachshon A et al. SARS-CoV-2 uses a multipronged strategy to impede host protein synthesis. Nature. 2021;594:240–5. 33979833 10.1038/s41586-021-03610-3

[bib29] Fisher T, Gluck A, Narayanan K et al. Parsing the role of NSP1 in SARS-CoV-2 infection. Cell Rep. 2022;39:110954. 35671758 10.1016/j.celrep.2022.110954 PMC9133101

[bib30] Foolchand A, Mazaleni S, Ghazi T et al. A review: highlighting the links between epigenetics, COVID-19 infection, and vitamin D. Int J Mol Sci. 2022;23:12292. 36293144 10.3390/ijms232012292 PMC9603374

[bib31] Friedman MJ, Lee H, Kwon YC et al. Dynamics of viral and host 3D genome structure upon infection. J Microbiol Biotechnol. 2022;32:1515–26. 36398441 10.4014/jmb.2208.08020 PMC9843816

[bib32] Fritz AJ, El Dika M, Toor RH et al. Epigenetic-mediated regulation of gene expression for biological control and cancer: cell and tissue structure, function, and phenotype. Results Probl Cell Differ. 2022;70:339–73. 36348114 10.1007/978-3-031-06573-6_12 PMC9753575

[bib33] Gan H, Serra-Cardona A, Hua X et al. The Mcm2-Ctf4-polalpha axis facilitates parental histone H3-H4 transfer to lagging strands. Mol Cell. 2018;72:140–51. 30244834 10.1016/j.molcel.2018.09.001 PMC6193272

[bib34] Ghaleh SS, Rahimian K, Mahmanzar M et al. SARS-CoV-2 non-structural protein 1(NSP1) mutation virulence and natural selection: evolutionary trends in the six continents. Virus Res. 2023;323:199016. 36473671 10.1016/j.virusres.2022.199016 PMC9721189

[bib35] Giroux NS, Ding S, McClain MT et al. Differential chromatin accessibility in peripheral blood mononuclear cells underlies COVID-19 disease severity prior to seroconversion. Sci Rep. 2022;12:11714. 35810186 10.1038/s41598-022-15668-8 PMC9271053

[bib36] Gomez GN, Abrar F, Dodhia MP et al. SARS coronavirus protein nsp1 disrupts localization of Nup93 from the nuclear pore complex. Biochem Cell Biol. 2019;97:758–66. 30943371 10.1139/bcb-2018-0394

[bib37] Gordon DE, Hiatt J, Bouhaddou M et al. Comparative host-coronavirus protein interaction networks reveal pan-viral disease mechanisms. Science. 2020a;370. 10.1126/science.abe9403. PMC7808408 33060197

[bib38] Gordon DE, Jang GM, Bouhaddou M et al. A SARS-CoV-2 protein interaction map reveals targets for drug repurposing. Nature. 2020b;583:459–68. 32353859 10.1038/s41586-020-2286-9 PMC7431030

[bib39] Gurung D, Danielson JA, Tasnim A et al. Proline isomerization: from the chemistry and biology to therapeutic opportunities. Biology. 2023;12:1008. 37508437 10.3390/biology12071008 PMC10376262

[bib40] Ho HH, Ivashkiv LB. Role of STAT3 in type I interferon responses. Negative regulation of STAT1-dependent inflammatory gene activation. J Biol Chem. 2006;281:14111–8. 16571725 10.1074/jbc.M511797200

[bib41] Ho JSY, Mok BW, Campisi L et al. TOP1 inhibition therapy protects against SARS-CoV-2-induced lethal inflammation. Cell. 2021;184:2618–32. 33836156 10.1016/j.cell.2021.03.051 PMC8008343

[bib42] Hornbeck PV, Zhang B, Murray B et al. PhosphoSitePlus, 2014: mutations, PTMs and recalibrations. Nucleic Acids Res. 2015;43:D512–20. 25514926 10.1093/nar/gku1267 PMC4383998

[bib43] Hu B, Guo H, Zhou P et al. Characteristics of SARS-CoV-2 and COVID-19. Nat Rev Microbiol. 2021;19:141–54. 33024307 10.1038/s41579-020-00459-7 PMC7537588

[bib44] Hu X, Dutta P, Tsurumi A et al. Unphosphorylated STAT5A stabilizes heterochromatin and suppresses tumor growth. Proc Natl Acad Sci USA. 2013;110:10213–8. 23733954 10.1073/pnas.1221243110 PMC3690839

[bib45] Huang C, Lokugamage KG, Rozovics JM et al. SARS coronavirus nsp1 protein induces template-dependent endonucleolytic cleavage of mRNAs: viral mRNAs are resistant to nsp1-induced RNA cleavage. PLoS Pathog. 2011;7:e1002433. 22174690 10.1371/journal.ppat.1002433 PMC3234236

[bib46] Huckriede J, de Vries F, Hultstrom M et al. Histone H3 cleavage in severe COVID-19 ICU patients. Front Cell Infect Microbiol. 2021;11:694186. 34568088 10.3389/fcimb.2021.694186 PMC8461091

[bib47] Huoman J, Sayyab S, Apostolou E et al. Epigenetic rewiring of pathways related to odour perception in immune cells exposed to SARS-CoV-2 in vivo and in vitro. Epigenetics. 2022;17:1875–91. 35758003 10.1080/15592294.2022.2089471 PMC9665140

[bib48] Jiang F, Hedaya OM, Khor E et al. RNA binding protein PRRC2B mediates translation of specific mRNAs and regulates cell cycle progression. Nucleic Acids Res. 2023;51:5831–46. 37125639 10.1093/nar/gkad322 PMC10287950

[bib49] Kamitani W, Huang C, Narayanan K et al. A two-pronged strategy to suppress host protein synthesis by SARS coronavirus Nsp1 protein. Nat Struct Mol Biol. 2009;16:1134–40. 19838190 10.1038/nsmb.1680 PMC2784181

[bib50] Kamitani W, Narayanan K, Huang C et al. Severe acute respiratory syndrome coronavirus nsp1 protein suppresses host gene expression by promoting host mRNA degradation. Proc Natl Acad Sci USA. 2006;103:12885–90. 16912115 10.1073/pnas.0603144103 PMC1568942

[bib51] Kandwal S, Fayne D. Genetic conservation across SARS-CoV-2 non-structural proteins—insights into possible targets for treatment of future viral outbreaks. Virology. 2023;581:97–115. 36940641 10.1016/j.virol.2023.02.011 PMC9999249

[bib52] Karousis ED. The art of hijacking: how Nsp1 impacts host gene expression during coronaviral infections. Biochem Soc Trans. 2024;52:481–90. 38385526 10.1042/BST20231119 PMC10903449

[bib53] Kgatle MM, Lawal IO, Mashabela G et al. COVID-19 is a multi-organ aggressor: epigenetic and clinical marks. Front Immunol. 2021;12:752380. 34691068 10.3389/fimmu.2021.752380 PMC8531724

[bib54] Kilkenny ML, Veale CE, Guppy A et al. Structural basis for the interaction of SARS-CoV-2 virulence factor nsp1 with DNA polymerase alpha-primase. Protein Sci. 2022;31:333–44. 34719824 10.1002/pro.4220 PMC8661717

[bib55] Kim SJ, Fernandez-Martinez J, Nudelman I et al. Integrative structure and functional anatomy of a nuclear pore complex. Nature. 2018;555:475–82. 29539637 10.1038/nature26003 PMC6022767

[bib56] Konigsberg IR, Barnes B, Campbell M et al. Host methylation predicts SARS-CoV-2 infection and clinical outcome. Commun Med. 2021;1:42. 10.1038/s43856-021-00042-y PMC8767772 35072167

[bib57] Lachner M, O’Carroll D, Rea S et al. Methylation of histone H3 lysine 9 creates a binding site for HP1 proteins. Nature. 2001;410:116–20. 11242053 10.1038/35065132

[bib58] Lapointe CP, Grosely R, Johnson AG et al. Dynamic competition between SARS-CoV-2 NSP1 and mRNA on the human ribosome inhibits translation initiation. Proc Natl Acad Sci USA. 2021;118. 10.1073/pnas.2017715118. PMC8017934 33479166

[bib59] Lechner MS, Schultz DC, Negorev D et al. The mammalian heterochromatin protein 1 binds diverse nuclear proteins through a common motif that targets the chromoshadow domain. Biochem Biophys Res Commun. 2005;331:929–37. 15882967 10.1016/j.bbrc.2005.04.016

[bib60] Lee JD, Menasche BL, Mavrikaki M et al. Differences in syncytia formation by SARS-CoV-2 variants modify host chromatin accessibility and cellular senescence via TP53. Cell Rep. 2023;42:113478. 37991919 10.1016/j.celrep.2023.113478 PMC10785701

[bib61] Lee JG, Huang W, Lee H et al. Characterization of SARS-CoV-2 proteins reveals Orf6 pathogenicity, subcellular localization, host interactions and attenuation by Selinexor. Cell Biosci. 2021;11:58. 33766124 10.1186/s13578-021-00568-7 PMC7993076

[bib62] Leppkes M, Knopf J, Naschberger E et al. Vascular occlusion by neutrophil extracellular traps in COVID-19. EBioMedicine. 2020;58:102925. 32745993 10.1016/j.ebiom.2020.102925 PMC7397705

[bib63] Li G, Tang Z, Fan W et al. Atlas of interactions between SARS-CoV-2 macromolecules and host proteins. Cell Insight. 2023;2:100068. 37192911 10.1016/j.cellin.2022.100068 PMC9670597

[bib64] Li Q, Zhang Z. Linking DNA replication to heterochromatin silencing and epigenetic inheritance. ABBS. 2012;44:3–13. 22194009 10.1093/abbs/gmr107 PMC3244654

[bib65] Li S, Ma F, Yokota T et al. Metabolic reprogramming and epigenetic changes of vital organs in SARS-CoV-2-induced systemic toxicity. JCI Insight. 2021a;6. 10.1172/jci.insight.145027. PMC7934846 33284134

[bib66] Li S, Wu B, Ling Y et al. Epigenetic landscapes of single-cell chromatin accessibility and transcriptomic immune profiles of T cells in COVID-19 patients. Front Immunol. 2021b;12:625881. 33717140 10.3389/fimmu.2021.625881 PMC7943924

[bib67] Li WX. Canonical and non-canonical JAK-STAT signaling. Trends Cell Biol. 2008;18:545–51. 18848449 10.1016/j.tcb.2008.08.008 PMC3082280

[bib68] Li Z, Hua X, Serra-Cardona A et al. DNA polymerase alpha interacts with H3-H4 and facilitates the transfer of parental histones to lagging strands. Sci Adv. 2020;6:eabb5820. 32923642 10.1126/sciadv.abb5820 PMC7449674

[bib69] Liu J, Li Y, Liu Q et al. SARS-CoV-2 cell tropism and multiorgan infection. Cell Discov. 2021;7:17. 33758165 10.1038/s41421-021-00249-2 PMC7987126

[bib70] Liu J, Ren X, Yin H et al. Mutation in the catalytic subunit of DNA polymerase alpha influences transcriptional gene silencing and homologous recombination in *Arabidopsis*. Plant J. 2010;61:36–45. 19769574 10.1111/j.1365-313X.2009.04026.x

[bib71] Lokugamage KG, Narayanan K, Huang C et al. Severe acute respiratory syndrome coronavirus protein nsp1 is a novel eukaryotic translation inhibitor that represses multiple steps of translation initiation. J Virol. 2012;86:13598–608. 23035226 10.1128/JVI.01958-12 PMC3503042

[bib72] Maeda R, Tachibana M. HP1 maintains protein stability of H3K9 methyltransferases and demethylases. EMBO Rep. 2022;23:e53581. 35166421 10.15252/embr.202153581 PMC8982598

[bib73] Majoros A, Platanitis E, Kernbauer-Holzl E et al. Canonical and non-canonical aspects of JAK-STAT signaling: lessons from interferons for cytokine responses. Front Immunol. 2017;8:29. 28184222 10.3389/fimmu.2017.00029 PMC5266721

[bib74] Malone B, Urakova N, Snijder EJ et al. Structures and functions of coronavirus replication-transcription complexes and their relevance for SARS-CoV-2 drug design. Nat Rev Mol Cell Biol. 2022;23:21–39. 34824452 10.1038/s41580-021-00432-z PMC8613731

[bib75] Maurina SF, O'Sullivan JP, Sharma G et al. An evolutionarily conserved strategy for ribosome binding and host translation inhibition by beta-coronavirus non-structural protein 1. J Mol Biol. 2023;435:168259. 37660941 10.1016/j.jmb.2023.168259 PMC10543557

[bib76] Meyer T, Gavenis K, Vinkemeier U. Cell type-specific and tyrosine phosphorylation-independent nuclear presence of STAT1 and STAT3. Exp Cell Res. 2002;272:45–55. 11740864 10.1006/excr.2001.5405

[bib77] Miao Z, Tidu A, Eriani G et al. Secondary structure of the SARS-CoV-2 5'-UTR. RNA Biol. 2021;18:447–56. 32965173 10.1080/15476286.2020.1814556 PMC7544965

[bib78] Nakayama J, Allshire RC, Klar AJ et al. A role for DNA polymerase alpha in epigenetic control of transcriptional silencing in fission yeast. EMBO J. 2001;20:2857–66. 11387218 10.1093/emboj/20.11.2857 PMC125490

[bib79] Narayanan K, Huang C, Lokugamage K et al. Severe acute respiratory syndrome coronavirus nsp1 suppresses host gene expression, including that of type I interferon, in infected cells. J Virol. 2008;82:4471–9. 18305050 10.1128/JVI.02472-07 PMC2293030

[bib80] Narayanan K, Ramirez SI, Lokugamage KG et al. Coronavirus nonstructural protein 1: common and distinct functions in the regulation of host and viral gene expression. Virus Res. 2015;202:89–100. 25432065 10.1016/j.virusres.2014.11.019 PMC4444399

[bib81] Nasheuer HP, Onwubiko NO. Lagging strand initiation processes in DNA replication of eukaryotes-strings of highly coordinated reactions governed by multiprotein complexes. Genes. 2023;14:1012. 37239371 10.3390/genes14051012 PMC10218536

[bib82] Newell-Price J, Clark AJ, King P. DNA methylation and silencing of gene expression. Trends Endocrinol Metabol. 2000;11:142–8. 10.1016/s1043-2760(00)00248-4 10754536

[bib83] Nikesjo F, Sayyab S, Karlsson L et al. Defining post-acute COVID-19 syndrome (PACS) by an epigenetic biosignature in peripheral blood mononuclear cells. Clin Epigenet. 2022;14:172. 10.1186/s13148-022-01398-1 PMC9748378 36517875

[bib84] Noguera-Castells A, Parra J, Davalos V et al. Epigenetic fingerprint of the SARS-CoV-2 infection in the lung of lethal COVID-19. Chest. 2024;165:820–4. 37914026 10.1016/j.chest.2023.10.032

[bib85] Padeken J, Methot SP, Gasser SM. Establishment of H3K9-methylated heterochromatin and its functions in tissue differentiation and maintenance. Nat Rev Mol Cell Biol. 2022;23:623–40. 35562425 10.1038/s41580-022-00483-w PMC9099300

[bib86] Pellegrini L. The Pol alpha-primase complex. Subcell Biochem. 2012;62:157–69. 22918585 10.1007/978-94-007-4572-8_9

[bib87] Platanias LC. Mechanisms of type-I- and type-II-interferon-mediated signalling. Nat Rev Immunol. 2005;5:375–86. 15864272 10.1038/nri1604

[bib88] Pombo A, Dillon N. Three-dimensional genome architecture: players and mechanisms. Nat Rev Mol Cell Biol. 2015;16:245–57. 25757416 10.1038/nrm3965

[bib89] Pontelli MC, Castro IA, Martins RB et al. SARS-CoV-2 productively infects primary human immune system cells in vitro and in COVID-19 patients. J Mol Cell Biol. 2022;14. 10.1093/jmcb/mjac021. PMC9384834 35451490

[bib90] Prentice E, McAuliffe J, Lu X et al. Identification and characterization of severe acute respiratory syndrome coronavirus replicase proteins. J Virol. 2004;78:9977–86. 15331731 10.1128/JVI.78.18.9977-9986.2004 PMC514967

[bib91] Puray-Chavez M, LaPak KM, Schrank TP et al. Systematic analysis of SARS-CoV-2 infection of an ACE2-negative human airway cell. Cell Rep. 2021;36:109364. 34214467 10.1016/j.celrep.2021.109364 PMC8220945

[bib92] Rual JF, Venkatesan K, Hao T et al. Towards a proteome-scale map of the human protein-protein interaction network. Nature. 2005;437:1173–8. 16189514 10.1038/nature04209

[bib93] Salgueiro BA, Saramago M, Tully MD et al. SARS-CoV2 Nsp1 is a metal-dependent DNA and RNA endonuclease. Biometals. 2024. 10.1007/s10534-024-00596-z. PMC11473540 38538957

[bib94] Samavarchi-Tehrani P, Abdouni H, Knight JDR et al. A SARS-CoV-2–host proximity interactome. Biorxiv. 2020. 10.1101/2020.09.03.282103.

[bib95] Schubert K, Karousis ED, Jomaa A et al. SARS-CoV-2 Nsp1 binds the ribosomal mRNA channel to inhibit translation. Nat Struct Mol Biol. 2020;27:959–66. 32908316 10.1038/s41594-020-0511-8

[bib96] Shemesh M, Aktepe TE, Deerain JM et al. SARS-CoV-2 suppresses IFNbeta production mediated by NSP1, 5, 6, 15, ORF6 and ORF7b but does not suppress the effects of added interferon. PLoS Pathog. 2021;17:e1009800. 34437657 10.1371/journal.ppat.1009800 PMC8389490

[bib97] Shen XR, Geng R, Li Q et al. ACE2-independent infection of T lymphocytes by SARS-CoV-2. Sig Transduct Target Ther. 2022;7:83. 10.1038/s41392-022-00919-x PMC8914143 35277473

[bib98] Shi FS, Yu Y, Li YL et al. Expression profile and localization of SARS-CoV-2 nonstructural replicase proteins in infected cells. Microbiol Spectr. 2022;10:e0074422. 35730969 10.1128/spectrum.00744-22 PMC9431475

[bib99] Shi S, Calhoun HC, Xia F et al. JAK signaling globally counteracts heterochromatic gene silencing. Nat Genet. 2006;38:1071–6. 16892059 10.1038/ng1860 PMC3092431

[bib100] Shi S, Larson K, Guo D et al. Drosophila STAT is required for directly maintaining HP1 localization and heterochromatin stability. Nat Cell Biol. 2008;10:489–96. 18344984 10.1038/ncb1713 PMC3083919

[bib101] Shultz RW, Tatineni VM, Hanley-Bowdoin L et al. Genome-wide analysis of the core DNA replication machinery in the higher plants *Arabidopsis* and rice. Plant Physiol. 2007;144:1697–714. 17556508 10.1104/pp.107.101105 PMC1949880

[bib102] Simeoni M, Cavinato T, Rodriguez D et al. I(nsp1)ecting SARS-CoV-2-ribosome interactions. Commun Biol. 2021;4:715. 34112887 10.1038/s42003-021-02265-0 PMC8192748

[bib103] Simon AC, Zhou JC, Perera RL et al. A Ctf4 trimer couples the CMG helicase to DNA polymerase alpha in the eukaryotic replisome. Nature. 2014;510:293–7. 24805245 10.1038/nature13234 PMC4059944

[bib104] Smothers JF, Henikoff S. The HP1 chromo shadow domain binds a consensus peptide pentamer. Curr Biol. 2000;10:27–30. 10660299 10.1016/s0960-9822(99)00260-2

[bib105] Snijder EJ, Bredenbeek PJ, Dobbe JC et al. Unique and conserved features of genome and proteome of SARS-coronavirus, an early split-off from the coronavirus group 2 lineage. J Mol Biol. 2003;331:991–1004. 12927536 10.1016/S0022-2836(03)00865-9 PMC7159028

[bib106] Sosnowski P, Tidu A, Eriani G et al. Correlated sequence signatures are present within the genomic 5'UTR RNA and NSP1 protein in coronaviruses. RNA. 2022;28:729–41. 35236777 10.1261/rna.078972.121 PMC9014872

[bib107] Starokadomskyy P, Escala Perez-Reyes A, Burstein E. Immune dysfunction in mendelian disorders of POLA1 deficiency. J Clin Immunol. 2021;41:285–93. 33392852 10.1007/s10875-020-00953-w PMC7864891

[bib108] Starokadomskyy P, Gemelli T, Rios JJ et al. DNA polymerase-alpha regulates the activation of type I interferons through cytosolic RNA:DNA synthesis. Nat Immunol. 2016;17:495–504. 27019227 10.1038/ni.3409 PMC4836962

[bib109] Starokadomskyy P, Wilton KM, Krzewski K et al. NK cell defects in X-linked pigmentary reticulate disorder. JCI Insight. 2019;4. 10.1172/jci.insight.125688. PMC6948767 31672938

[bib110] Tanaka T, Kamitani W, DeDiego ML et al. Severe acute respiratory syndrome coronavirus nsp1 facilitates efficient propagation in cells through a specific translational shutoff of host mRNA. J Virol. 2012;86:11128–37. 22855488 10.1128/JVI.01700-12 PMC3457165

[bib111] Tardivat Y, Sosnowski P, Tidu A et al. SARS-CoV-2 NSP1 induces mRNA cleavages on the ribosome. Nucleic Acids Res. 2023;51:8677–90. 37503833 10.1093/nar/gkad627 PMC10484668

[bib112] Thoms M, Buschauer R, Ameismeier M et al. Structural basis for translational shutdown and immune evasion by the Nsp1 protein of SARS-CoV-2. Science. 2020;369:1249–55. 32680882 10.1126/science.abc8665 PMC7402621

[bib113] Thul PJ, Akesson L, Wiking M et al. A subcellular map of the human proteome. Science. 2017;356. 10.1126/science.aal3321. 28495876

[bib114] Tsai K, Cullen BR. Epigenetic and epitranscriptomic regulation of viral replication. Nat Rev Microbiol. 2020;18:559–70. 32533130 10.1038/s41579-020-0382-3 PMC7291935

[bib115] Tsai MH, Pai LM, Lee CK. Fine-tuning of type I interferon response by STAT3. Front Immunol. 2019;10:1448. 31293595 10.3389/fimmu.2019.01448 PMC6606715

[bib116] V'Kovski P, Kratzel A, Steiner S et al. Coronavirus biology and replication: implications for SARS-CoV-2. Nat Rev Microbiol. 2021;19:155–70. 33116300 10.1038/s41579-020-00468-6 PMC7592455

[bib117] Wang R, Lee JH, Kim J et al. SARS-CoV-2 restructures host chromatin architecture. Nat Microbiol. 2023;8:679–94. 36959507 10.1038/s41564-023-01344-8 PMC10116496

[bib118] Wathelet MG, Orr M, Frieman MB et al. Severe acute respiratory syndrome coronavirus evades antiviral signaling: role of nsp1 and rational design of an attenuated strain. J Virol. 2007;81:11620–33. 17715225 10.1128/JVI.00702-07 PMC2168762

[bib119] Wulf G, Finn G, Suizu F et al. Phosphorylation-specific prolyl isomerization: is there an underlying theme?. Nat Cell Biol. 2005;7:435–41. 15867923 10.1038/ncb0505-435

[bib120] Xia H, Cao Z, Xie X et al. Evasion of type I interferon by SARS-CoV-2. Cell Rep. 2020;33:108234. 32979938 10.1016/j.celrep.2020.108234 PMC7501843

[bib121] Xu K, Li J, Li WX. Simulation of STAT and HP1 interaction by molecular docking. Cell Signal. 2023;112:110925. 37839545 10.1016/j.cellsig.2023.110925

[bib122] Yang X, Rutkovsky AC, Zhou J et al. Characterization of altered gene expression and histone methylation in peripheral blood mononuclear cells regulating inflammation in COVID-19 patients. J Immunol. 2022;208:1968–77. 35379747 10.4049/jimmunol.2101099 PMC9012677

[bib123] Yang Y, Guo L, Chen L et al. Nuclear transport proteins: structure, function, and disease relevance. Sig Transduct Target Ther. 2023;8:425. 10.1038/s41392-023-01649-4 PMC10636164 37945593

[bib124] Yi SJ, Kim K. Histone tail cleavage as a novel epigenetic regulatory mechanism for gene expression. BMB Rep. 2018;51:211–8. 29540259 10.5483/BMBRep.2018.51.5.053 PMC5988574

[bib125] Yoshimoto FK. The proteins of severe acute respiratory syndrome coronavirus-2 (SARS CoV-2 or n-COV19), the cause of COVID-19. Protein J. 2020;39:198–216. 32447571 10.1007/s10930-020-09901-4 PMC7245191

[bib126] You M, Chen L, Zhang D et al. Single-cell epigenomic landscape of peripheral immune cells reveals establishment of trained immunity in individuals convalescing from COVID-19. Nat Cell Biol. 2021;23:620–30. 34108657 10.1038/s41556-021-00690-1 PMC9105401

[bib127] Yuan S, Balaji S, Lomakin IB et al. Coronavirus Nsp1: immune response suppression and protein expression inhibition. Front Microbiol. 2021;12:752214. 34659188 10.3389/fmicb.2021.752214 PMC8512706

[bib128] Yuan S, Peng L, Park JJ et al. Nonstructural protein 1 of SARS-CoV-2 is a potent pathogenicity factor redirecting host protein synthesis machinery toward viral RNA. Mol Cell. 2020;80:1055–66. 33188728 10.1016/j.molcel.2020.10.034 PMC7833686

[bib129] Zhang J, Cruz-Cosme R, Zhuang MW et al. A systemic and molecular study of subcellular localization of SARS-CoV-2 proteins. Sig Transduct Target Ther. 2020a;5:269. 10.1038/s41392-020-00372-8 PMC7670843 33203855

[bib131] Zhang L, Lu Q, Chang C. Epigenetics in health and disease. Adv Exp Med Biol. 2020b;1253:3–55. 32445090 10.1007/978-981-15-3449-2_1

[bib130] Zhang K, Miorin L, Makio T et al. Nsp1 protein of SARS-CoV-2 disrupts the mRNA export machinery to inhibit host gene expression. Sci Adv. 2021a;7:eabe7386. 33547084 10.1126/sciadv.abe7386 PMC7864571

[bib132] Zhang Y, Sun Z, Jia J et al. Overview of histone modification. Adv Exp Med Biol. 2021b;1283:1–16. 33155134 10.1007/978-981-15-8104-5_1

[bib133] Zheng J, Wang Y, Li K et al. Severe acute respiratory syndrome coronavirus 2-induced immune activation and death of monocyte-derived Human macrophages and dendritic cells. J Infect Dis. 2021;223:785–95. 33277988 10.1093/infdis/jiaa753 PMC7799009

[bib134] Zheng Y, Liu X, Le W et al. A human circulating immune cell landscape in aging and COVID-19. Protein Cell. 2020;11:740–70. 32780218 10.1007/s13238-020-00762-2 PMC7417788

[bib135] Zhou S, Zhang J, Xu J et al. An epigenome-wide DNA methylation study of patients with COVID-19. Ann Hum Genet. 2021;85:221–34. 34185889 10.1111/ahg.12440 PMC8441705

[bib136] Zhou Y, Wang TS. A coordinated temporal interplay of nucleosome reorganization factor, sister chromatin cohesion factor, and DNA polymerase alpha facilitates DNA replication. Mol Cell Biol. 2004;24:9568–79. 15485923 10.1128/MCB.24.21.9568-9579.2004 PMC522230

[bib137] Zimmerman MA, Rahman NT, Yang D et al. Unphosphorylated STAT1 promotes sarcoma development through repressing expression of Fas and bad and conferring apoptotic resistance. Cancer Res. 2012;72:4724–32. 22805310 10.1158/0008-5472.CAN-12-1347 PMC3564959

